# Melatonin Alleviates Copper Toxicity via Improving ROS Metabolism and Antioxidant Defense Response in Tomato Seedlings

**DOI:** 10.3390/antiox11040758

**Published:** 2022-04-11

**Authors:** Tao Zhang, Yong Wang, Xiaojing Ma, Zhaopeng Ouyang, Lei Deng, Shunshan Shen, Xiaoxing Dong, Nanshan Du, Han Dong, Zhixin Guo, Geng Meng, Fengzhi Piao, Kaile Sun

**Affiliations:** 1 College of Horticulture, Henan Agricultural University, Zhengzhou 450002, China; zhangtao3375@163.com (T.Z.); yongwang@henau.edu.cn (Y.W.); maxiaojing4869@163.com (X.M.); ouyangzhaopeng@yeah.net (Z.O.); denglei9845@163.com (L.D.); wuxian_mige@163.com (X.D.); fangshan711@163.com (N.D.); 440069@henau.edu.cn (H.D.); guozhixin666@163.com (Z.G.); 2 College of Plant Protection, Henan Agricultural University, Zhengzhou 450002, China; shen0426@163.com

**Keywords:** heavy metal (HM), copper stress (CS), tomato, melatonin (MT), reactive oxygen species (ROS), antioxidants

## Abstract

The excessive accumulation of copper (Cu^2+^) has become a threat to worldwide crop production. Recently, it was revealed that melatonin (MT) could play a crucial role against heavy metal (HM) stresses in plants. However, the underlying mechanism of MT function acted upon by Cu^2+^ stress (CS) has not been substantiated in tomatoes. In the present work, we produced MT-rich tomato plants by foliar usage of MT, and MT-deficient tomato plants by employing a virus-induced gene silencing methodology and exogenous foliar application of MT synthesis inhibitor para-chlorophenylalanine (pCPA). The obtained results indicate that exogenous MT meaningfully alleviated the dwarf phenotype and impeded the reduction in plant growth caused by excess Cu^2+^. Furthermore, MT effectively restricted the generation of reactive oxygen species (ROS) and habilitated cellular integrity by triggering antioxidant enzyme activities, especially via CAT and APX, but not SOD and POD. In addition, MT increased nonenzymatic antioxidant activity, including FRAP and the GSH/GSSG and ASA/DHA ratios. MT usage improved the expression of several defense genes (*CAT*, *APX*, *GR* and *MDHAR*) and MT biosynthesis-related genes (*TDC*, *SNAT* and *COMT*). Taken together, our results preliminarily reveal that MT alleviates Cu^2+^ toxicity via ROS scavenging, enhancing antioxidant capacity when subjected to excessive Cu^2+^. These results build a solid foundation for developing new insights to solve problems related to CS.

## 1. Introduction

Heavy metal (HM) pollution is becoming a serious problem in the environment due to increasing activities from industrial, agricultural, and urban areas, which contaminate crops, resulting in a decrease in plant yield [1]. In addition, it causes a decrease in the quality of the produced food and negatively affects the health of living organisms [2]. Copper (Cu^2+^) is regarded as an essential micronutrient for plant growth and was first identified as such in the 1930s [3]. It is an important cofactor in proteins that are related to electron transfer reactions in plants [4]. However, extra Cu^2+^ can cause damage to physiological, biochemical, and growth processes in plants by inducing oxidative stress. In previous studies, Cu-induced toxicity included a reduction in leaf area and growth along with chlorosis [5], reduced photosynthesis capacity and grain yield [6], a reduction in the elongation of roots and shoots [7], and hindering the uptake of other necessary mineral elements [8]. Therefore, Cu^2+^ can be beneficial for plants at appropriate amounts, but toxic with extreme accumulation. Recently, Cu^2+^ pollution has been on the rise due to the increasing use of pesticides and fertilizers on crops in protected cultivation. Therefore, it will be of great value to develop more approaches to elucidate Cu^2+^ stress (CS).

Under the action of stress, reactive oxygen species (ROS) are generated in plants, causing oxidative stress, resulting in damage to other important molecules in plants; this is the generally accepted explanation for HM toxicity [9]. To adapt to adverse changes in the environment, plants have developed several distinct strategies to cope with various stresses, including Cu^2+^ toxicity. Usually, there are two strategies for plants to resist HMs: avoidance and tolerance. The avoidance strategy is to reduce the uptake of HMs by affecting the mobility of HMs within root exudates, as well as to sequester and compartmentalize extra Cu^2+^ in the roots, apoplast and vacuole, therefore avoiding the transportation of HMs to sensitive tissues [10,11,12,13,14]. The tolerance strategy is to remove the metal-induced ROS through chelating Cu^2+^ with other substances, osmotic regulation, and activating the antioxidant systems [15,16].

Melatonin (*N*-acetyl-5-methoxytryptamine; MT) was first discovered as a neurohormone originating in the bovine pineal gland in 1958 [17]. In the 1990s, MT was simultaneously found by researchers in higher plants [18,19]; since then, the number of studies of MT in plants has increased significantly, and there is more and more information known regarding the biosynthetic and possible degradation pathways of MT, as well as the molecular mechanisms related to its various functions in plants. The excellent properties of MT as an antioxidative molecule have been widely studied with regard to plant growth and development, in addition to numerous biotic and abiotic stress responses [20,21,22,23,24,25]. To be specific, MT acts as an effective free radical scavenger against abiotic stress, including low temperatures [21], drought [26], UV irradiation and herbicides [25], and salinity [27]. In addition, there have been studies on how MT reduces the toxicity of HM in plants. In radish plants, MT increased the activities of HM chelators and transporters, which resulted in cadmium tolerance [15]. Exogenous MT application also alleviates lead and nickel phytotoxicity [28,29]. In cucumber, MT was reported to improve copper sequestration and ROS scavenging under copper toxicity [30]. Tomato, a major crop plant, is faced with serious Cu^2+^ toxicity. However, the mechanisms for how MT alleviates Cu^2+^ toxicity in tomato have not been scrutinized.

To realize the function of MT in response to CS, we conducted a set of experiments in tomato at the physiological, biochemical and molecular levels. The effects of exogenous MT subjected to additional Cu^2+^ on plant growth, generation of ROS, endogenous MT and Cu concentrations, and enzymatic and nonenzymatic antioxidant capacity and gene expression were determined. Additionally, a virus-induced gene silencing (VIGS) approach and the exogenous foliar application of MT synthesis inhibitor pCPA were exploited to perform MT-deficient treatment under CS. The obtained results revealed the role of MT in the presence of CS through regulating ROS production and enhancing the antioxidant defense system. To the best of our knowledge, this is the first investigation examining the mechanisms of how MT responds to CS in tomato.

## 2. Materials and Methods

### 2.1. Plant Materials and Growth Conditions

Tomato seeds (*Solanum lycopersicum* L. cv. Condine Red) were placed in Petri dishes that contained two layers of moistened filter paper, and then left in the dark for 30 h at 28 ± 1 °C. After germination, the seeds were dotted in a growth medium, which contained peat, vermiculite and perlite (3:1:1, *v*/*v*). After the secondary true leaves were completely grown, seedlings were shifted to plastic containers (21.5 cm × 16 cm × 14 cm) containing half-strength Hoagland’s nutrient solution for various treatments. Subsequently, the plants were cultivated at a temperature of 28/20 ± 1 °C (day/night), a midpoint humidity around 70% to 80%, and 14/10 h (day/night) photoperiods with a photosynthetic photon flux density of 200 µmol m^−2^ s^−1^.

When the third true leaves were completely grown, seedlings were treated with an ordinary culture solution (control; CK), a culture solution containing 100 µM Cu^2+^ (supplied as CuSO_4_), a culture solution with folia application of 100 µM MT, and a culture solution containing Cu^2+^ with folia application of 100 µM (Cu + MT) or MT synthesis inhibitor pCPA (Cu + pCPA). Plants were sprayed with 100 µM MT on the leaves at 18:00 (approximately 5 mL per plant) on the first day. After 12 h, 50 µM Cu^2+^ was given with CuSO_4_ (analytical grade) in the solution of the Cu-treated plants. Subsequently, plants were sprayed with 100 µM MT every day. To avoid devastating damage caused by a high concentration of Cu^2+^ on tomato seedlings, such as killing the plants, the dosage of Cu^2+^ was increased from 50 to 100 µM in this study. The dosages of 100 µM Cu^2+^ and MT were chosen based on preliminary tests, and 100 µM pCPA were chosen based on a previous study (data not shown). The culture solution of each treatment changed every 3 d. Each box contained six seedlings. For each treatment, 36 seedlings were used. After 14 days of Cu treatment, the second and third leaves (from the top) were collected for further analysis.

### 2.2. Evaluation of Morphological and Photosynthesis Parameters

After fourteen days of treatment, the phenotypes were analyzed, including plant height and fresh and dry weight (FW and DW). Triphenyl tetrazolium chloride (TTC), a chemical compound, was employed to assess fine-root vitality [31]. The shoot and root DWs were obtained by oven-drying the samples at 80 °C for 48 h. To measure the chlorophyll content of leaves, fully unfolded fresh leaves from each treatment (0.2 g) were carefully ground into homogenate with 25 mL of 80% acetone. The supernatant was collected after centrifugation, and absorbance was read at 663 and 646 nm using a UV-1800 spectrophotometer. The concentration was calculated using the following formula [32].

Chlorophyll a (C_a_, mg/L) = 12.21A_663_ − 2.81A_646_

Chlorophyll b (C_b_, mg/L) = 20.13A_646_ − 5.03A_663_

Total Chlorophyll = C_a_+ C_b_

The chlorophyll content (mg/g·FW) was further obtained by the formula: C × V × *n*/W (C means concentration; V is the volume, namely, 0.025 L; *n* is the dilution ratio, namely, 1; W is the fresh weight of the sample, namely, 0.2 g).

### 2.3. Transmission Electron Microscope (TEM) Analysis

The secondary unfurled leaves were cropped to segments with lengths of 0.001 m. Subsequently, these were subjected to four percent glutaraldehyde for 12 h, and flushed three times with a 0.1 M sodium phosphate buffer. The resulting segments were then post-fixed in 1% osmium tetroxide (OsO_4_) in darkness for 2 h and flushed three times via 0.1 M sodium phosphate buffer again. The samples were dehydrated via a graded ethanol series and were inserted in paraffin. A transmission electron microscope (Hitachi-7800) was exploited to photograph mesophyll cells.

### 2.4. Assessment of MDA, Relative Electrolyte Leakage and Histochemical Recognition of H_2_O_2_ and O_2_^•−^

Hydrogen peroxide (H_2_O_2_) and superoxide anions (O_2_^•−^) in plant tissue were reflected utilizing 3,3-diaminobenzidine (DAB) or the nitro blue tetrazolium (NBT) approach, respectively. H_2_O_2_ and O_2_^•−^ were shown as a brown or blue color as a result of DAB polymerization or NBT reduction.

The distribution of H_2_O_2_ was assessed by means of DAB staining in tomato leaves followed by the methods described below. The discs of tomato leaves were collected and immersed in a pipe full of DAB staining solution, which consisted of 0.1% DAB in 10 mM 2-(n-morpholinyl) ethanesulfonic acid (MES) buffer with pH 6.5 [33]. We kept the tube at room temperature until a dark brown color was visible (about 8 h). Then, we drained the staining solution from the test tubes. To remove the chlorophyll for proper visualization of the stain, the leaf discs from the previous step were immersed in absolute ethanol and heated in a boiling water bath for 5 min. Finally, we poured out the ethanol and gently rinsed the leaf discs three times with distilled water. The presence and distribution of H_2_O_2_ could thus be visualized and photographed. The O_2_^•−^ content was determined by NBT staining. Tomato leaf discs were collected and immersed in NBT solution, containing 0.1% NBT in 50 mM potassium phosphate and 10 mM sodium azide with pH 6.4. The samples were left in the NBT staining solution in the dark at 28 °C until blue dots were displayed on the leaves [34]. The remaining steps were the same as described above to remove the chlorophyll.

The content of H_2_O_2_ was measured with the trichloroacetic acid (TCA) method according to Velikova et al. [35]. To be specific, 0.5 g samples were used and mixed with 0.1% TCA. After centrifugation at 12,000× *g* for 15 min, the supernatant was used for the measurement of absorbance at 390 nm using a microplate reader.

The level of MDA was measured as displayed in an earlier work [36]. Briefly, we weighed out approximately 0.3 g of the sample and ground it into a homogenate on ice with 3 mL of the extract solution, which contained 0.1% (*w*/*v*) trichloroacetic acid (TCA). Then, 1 mL of supernatant was obtained and added into a 4 mL aliquot of 20% TCA containing 0.5% 2-thiobarbituric acid (TBA). MDA absorption was monitored at 450, 532 and 600 nm using a microplate reader.

For measurement of the relative electrolyte leakage (REL), the third totally expanded leaves from the plants were utilized. They were cut into 1 cm slices, briefly cleaned with water, and instantly positioned in a tube with water at 25 °C. After 2 h, the conductivity was measured [29].

### 2.5. Measurement of Enzymatic Antioxidant Compounds

In order to assess the enzymatic antioxidant activities, approximately 0.2 g of sample was weighed. Depending on the type of enzyme tested, samples were extracted and tested with the appropriate kit (Suzhou Michy Biomedical Technology Co., Ltd., Suzhou, China) according to the manufacturer’s instructions. Generally, extract solution (2 mL) was used to grind the sample into a homogenate in an ice bath. Subsequently, the homogenate was centrifuged at 12,000 rpm for 10 min at 4 °C, and the supernatant was taken and placed on ice for testing. Superoxide dismutase (SOD) activity was measured using the WST-8 method as described by Ukeda et al. [37]. Readings at 450 nm were collected with a microplate reader. Peroxidase (POD) activity was calculated using guaiacol as described previously [38]. The activity of catalase (CAT) was determined according to the methodology described previously [39]. Ascorbate peroxide (APX) activity was calculated using a spectrophotometer at 290 nm as described by Nakano and Asada [40].

### 2.6. Evaluation of Nonenzymatic Antioxidant Mixtures

For the assessment of total phenolic and flavonoid concentrations, a leaf sample with a weight of 200 mg was ground into a homogenate with 6 mL of 80% ethanol. Subsequently, the resulting mixture was centrifuged under the action of 12,000× *g* for 1/3 h at the temperature level given in Section 2.5, and the supernatant was collected for assessing the phenolic and flavonoid concentrations. Phenolic concentrations were determined by means of the Folin–Ciocalteu colorimetric approach based on Singleton and Rossi (1965) [41]. The amount of flavonoid in the leaves was then examined by implementing the methodologies explained by Jia et al. [42].

The ferric-reducing antioxidant power (FRAP) and 1,1-diphenyl-2-picrylhydrazyl (DPPH) assays were utilized to check the antioxidant capacity. The stock solutions for the FRAP test included 20 mM ferric chloride (FeCl_3_) solution, 300 mM acetate buffer (3.1 g of C_2_H_3_NaO_2_.3H_2_O and 16 mL of C_2_H_4_O_2_), pH 3.6, and 10 mM 2,4,6-tripyridyl-s-triazine (TPTZ) in 40 mM hydrochloric acid (HCl). Then, a fresh working solution was obtained by mixing 2.5 mL of TPTZ solution, 2.5 mL of FeCl_3_.6H_2_O solution and 2.5 mL of acetate buffer, and the solution was warmed to 37 °C before use. The stock solution for the DPPH assay was prepared by adding 24 mg of DPPH into 100 mL of methanol, and then kept at 20 °C until needed. The working solution was prepared by mixing 10 mL of stock solution with 45 mL of methanol. Both FRAP and DPPH were measured within 150 µL leaf extracts by measuring the change in absorbance at 593 and 515 nm, respectively, as described by a previous study [43].

The ascorbic acid (AsA) and dehydroascorbate (DHA) levels were extracted using 6% (*v*/*v*) HClO_4_ and detected by measuring the change in absorbance at 534 and 265 nm, respectively, using the microplate reader [44].

Additionally, 5% (*v*/*v*) sulfosalicylic acid was prepared for the extraction of oxidized glutathione (GSSG) and reduced glutathione (GSH). The contents of GSSG and GSH were evaluated at 412 nm using the methodology suggested by Griffith [45].

The free proline content was determined using commercial kits (Suzhou Michy Biomedical Technology Co., Ltd.). Briefly, 0.1 g sample was weighed and made into homogenate with 1 mL of extract solution (mainly sulfosalicylic acid). It was placed in a 90 °C water bath for 10 min and extracted via shaking. After being centrifuged at 10,000 rpm for 10 min at 25 °C, the supernatant was taken and reacted with acidic ninhydrin solution to form a red color, and then extracted with methylbenzene. The proline content was evaluated by monitoring the supernatant absorbance at 520 nm.

### 2.7. Measurement of Cu and MT Contents in Shoot and Root

For determination of the Cu content, the shoots and roots of samples were harvested and oven-dried to an unvarying weight at 65 °C. The dried samples were added into a polytetrafluoroethylene digestion tank containing 5 mL of HNO_3_ and 2 mL of H_2_O_2_. They were digested until clear, then diluted to 50 mL after filtering, and the endogenous Cu contents were determined using ICP-MS. The sample analyses were performed in triplicate.

The leaves and roots of the tomato seedlings were sampled and thereafter desiccated to an unvarying weight at 65 °C using an oven. Subsequently, Cu was extracted using nitric acid at 160 °C, and the concentrations were evaluated employing the ICP-MS. MT was tested exploiting the approach proposed by Pothinuch and Tongchitpakdee [46]. One gram of the sample was utilized, along with five milliliters of methanol. After the sample was ground into a homogenate with a grinder, it was ultrasonically derived in an ice-water bath for 1 h and centrifuged to obtain the supernatant. The supernatant was eluted via a 220 µm syringe filter by adding 1 mL of 80% methanol. Then, the supernatant was ready for analysis. The analysis conditions were as follows:

The chromatograph was the Waters2695 high performance liquid chromatograph (Agilent Technologies, Santa Clara, CA, USA) with a fluorescence detector, the excitation wavelength was 280 nm, and the emission wavelength was 340 nm; the chromatographic column was a reversed-phase column (Compass C18) (250 mm × 4.6 mm, 5 µm) at a temperature of 30 °C. Flow rate was 1.0 mL/min and injection volume was 10 µL. The mobile phase was water: methanol = 65:35 (*v*/*v*).

### 2.8. Virus-Induced Gene Silencing Constructs and Agrobacterium-Mediated Virus Infection

To examine the influence of endogenous MT, the VIGS approach was exploited to silence the *COMT1* gene of tomato. This gene (300 bp fragment) was appropriately PCR amplified from tomato cDNA by employing the forward and reverse primers with XbaI and KpnI restriction sites (Supplementary File S1). The TRV2 plasmid was processed with identical enzymes and ligated with the *COMT1* gene. The correct plasmid, after conducting the PCR test and sequencing, was transformed into the *Agrobacterium tumefaciens* GV3101. For the injection of *A. tumefaciens*, the wholly expanded cotyledonary leaves were infiltrated with *A. tumefaciens* taking the TRV1-TRV2 and TRV1-TRV2 objective genes in a 1:1 ratio, respectively. The empty TRV1 and TRV2 vectors and empty TRV1 with PDS were used as controls, respectively. The efficacy of the VIGS and inhibition of the MT biosynthesis were rationally validated via examination of the comparative manifestation of *COMT1*.

### 2.9. RNA Extraction and Gene Expression Assay via the Quantitative Real-Time PCR Approach

Tomato leaf tissues (100 mg) were employed for deriving the RNA utilizing the PureLink RNA Kit (Thermo Fisher Scientific, Waltham, MA, USA) following the manufacturer’s instructions. The quality and quantity of RNA samples were assessed on a NanoDrop 2000 spectrophotometer (Thermo Scientific, Waltham, MA, USA). For the purpose of producing cDNA, 1 µg of RNA was converted to cDNA employing MonScript™ RTIII All-in-One Mix with dsDNase (Monad, Wuhan, China) based on the manufacturer’s directions. The gene-specific primers were developed following prior analyses [29,47], and actin was used as an interior controller (Supplementary Table S1). Applied Biosystem SYBR (Thermo Fisher Scientific, MA, USA) was utilized to prepare Real-Time PCR (qRT-PCR), and the cycle conditions were: 95 °C for 3 min, followed by 40 cycles at 95 °C for 10 s and 60 °C for 30 s. The relative transcript levels were evaluated by Shen et al. [48].

### 2.10. Statistical Analysis

All tests in the present study were replicated three times, and the data are shown as means ± standard deviations (SDs). All the data were statically examined employing SPSS 20.0 package software. One-way analysis of variance (ANOVA) was conducted, and mean discrepancies were assessed by Tukey’s honest significant difference (HSD) test at *p* < 0.05.

## 3. Results

### 3.1. Influence of MT on the Growth of Tomato Plants Subjected to CS

**Figure 1:**
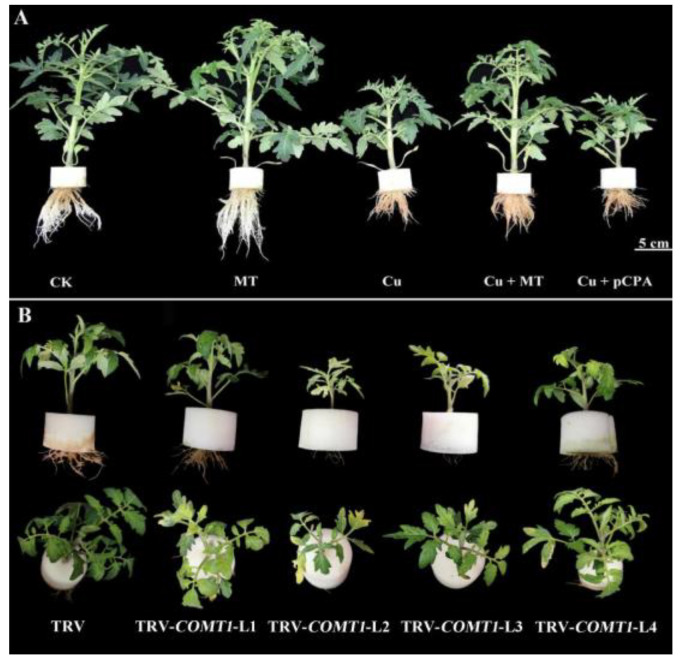
(**A**) Phenotypes after 14 d of Cu^2+^ and melatonin (MT) or pCPA treatment. CK, ordinary culture solution; Cu, culture solution containing 0.1 mM Cu; MT, ordinary culture with folia application of 0.1 mM MT; Cu + MT, ordinary culture solution containing 0.1 mM Cu with folia application of 0.1 mM MT; Cu + pCPA, ordinary culture solution containing 0.1 mM Cu with 0.1 mM MT synthesis inhibitor pCPA. (**B**) Influences of *COMT1* silencing acted upon by the Cu stress on plant phenotype.

To investigate the role of MT in tomato in the presence of Cu^2+^ toxicity, tomato seedlings were grown in CK, Cu, MT, Cu + MT and Cu + pCPA treatments. The choice of Cu and MT concentrations for testing was in accordance with our preliminary testing in tomatoes, which concluded that 0.1 mM Cu^2+^ induced substantial inhibition of plant growth without killing plants, and 0.1 mM MT application to Cu-stressed plants considerably improved such destructive symptoms (data not shown). As demonstrated in Figure 1A and Supplementary Figure S1, seedlings treated with Cu^2+^ showed a dwarf phenotype, with small and rolled leaves at the top. This is further verified by the *COMT1* silencing, which resulted in a dwarf phenotype with small yellow leaves and brown-colored roots as a result of being subjected to CS (see Figure 1B). Root development was significantly inhibited by Cu treatment. The roots in the Cu treatment group were brown in color, fewer in number and shorter than those of CK and MT (see Figure 1A,B). By comparison, MT increased the number and length of roots.

The Cu toxicity on tomato plants was intensified by exogenous foliar application of pCPA. Cu + pCPA could lessen the plant height, shoot and root FW and DW, root vigor (TTC), and total chlorophyll by 32.53%, 40.19% and 35.66%, 42.99% and 37.5%, 56.1% and 7.5% compared to Cu, respectively (see Table 1). Phytotoxicity was decreased by exogenous MT, which attempted to mitigate the influence of Cu by improving the factors mentioned above by 32.96%, 39.76% and 88.93%, 31.75% and 37.5%, 330% and 78.75%, respectively, in the Cu + MT plants compared with the Cu-treated plants (see Table 1). Overall, root vigor was the most considerably damaged due to CS, and MT application could diminish such damage.

### 3.2. Influence of MT on Ultrastructure Alterations in Mesophyll Cells and Chloroplasts of Tomato Leaves Subjected to CS

**Figure 2:**
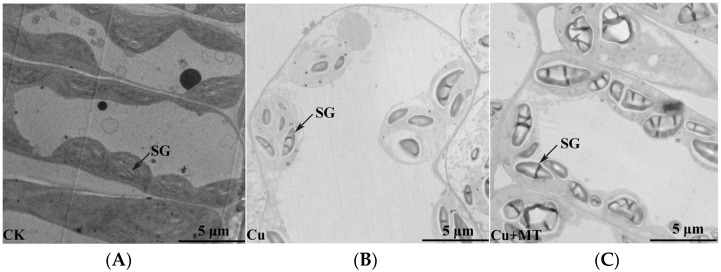
Transmission electron microscopy (TEM) of mesophyll cells of the second leaf from the top. (**A**) TEM structure of control, (**B**) Cu treatment, (**C**) MT+Cu treatment. CK, ordinary culture solution; Cu, culture solution containing 0.1 mM Cu; MT, ordinary culture with folia application of 0.1 mM MT; Cu + MT, ordinary culture solution containing 0.1 mM Cu with folia application of 0.1 mM MT; Cu + pCPA, ordinary culture solution containing 0.1 mM Cu with 0.1 mM MT synthesis inhibitor pCPA. Here, SG means starch granule.

To attain a better realization of how MT influenced the structures of cells and organelles subjected to CS, we inspected the leaves of the tomato seedlings via transmission electron microscopy (TEM) (see Figure 2). The ultrastructure of whole mesophyll cells and chloroplasts of the second leaf from the top of variously treated plants revealed many modifications to the cells’ dimensions and chloroplast formation (see Figure 2). In the CK, tomato seedlings had a regular cell form and chloroplasts demonstrated regular ellipsoidal forms and organized in an individual row in the vicinity of the cell wall (see Figure 2A). CS caused cell plasmolysis and morphological disorders. The chloroplasts no longer had ordinary ellipsoidal forms; some of the chloroplasts began to change from the original ellipsoidal shape to a spherical shape (see Figure 2B). In contrast to the control plants, tiny starch granules in chloroplasts were detected. After application of MT to the CS-based plants, the chloroplast’s form changed to an ellipsis or spindle shape, and the amount of starch granules in the chloroplasts of tomato plants was evidently amplified (see Figure 2C).

### 3.3. MT and Copper Content Modifications in MT and pCPA Treated Tomato Leaves

**Figure 3:**
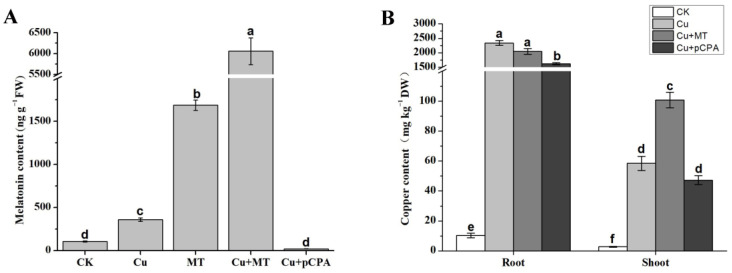
Effects of exogenous application of MT on roots and shoots in different treatments. (**A**) MT concentration; (**B**) Copper concentration. CK, ordinary culture solution; Cu, culture solution containing 0.1 mM Cu; MT, ordinary culture with folia application of 0.1 mM MT; Cu + MT, ordinary culture solution containing 0.1 mM Cu with folia application of 0.1 mM MT; Cu + pCPA, ordinary culture solution containing 0.1 mM Cu with 0.1 mM MT synthesis inhibitor pCPA. The various letters (a, b, c, d, e, f) specify considerable discrepancies for *p* values lower than 0.05 based on Duncan’s multiple scope tests.

In order to investigate whether exogenous application of MT could affect the endogenous generation of MT and Cu uptake, we examined the endogenous concentration of MT and Cu in the roots and shoots of tomato seedlings (see Figure 3). The MT content was low in CK. However, it increased to 360 ng/g FW and 1688 ng/g FW with standalone Cu and MT treatment, respectively, which indicates that CS could induce MT production slightly, and exogenous MT application could highly induce endogenous MT production (see Figure 3A). MT content was highest in the leaves of plant under MT+Cu treatment. Compared with other Cu-treated plants, Cu + pCPA-treated plants had lower MT content, demonstrating that MT was competently eliminated in tomato plants (see Figure 3A). The addition of Cu^2+^ yielded a considerable increase in the amount of Cu^2+^ within the shoots and roots of the tomato seedlings; however, the accumulation of Cu^2+^ was considerably higher in the roots than in the shoots (see Figure 3B). The amount of Cu^2+^ within the roots of Cu + MT-treated plants was not statistically different from, but slightly higher than that in the shoots of Cu-treated plants, which demonstrates that MT could not influence Cu uptake but did alleviate Cu transport (see Figure 3B). Furthermore, there was a slight reduction in Cu uptake in Cu + pCPA-treated plants compared with Cu-treated plants, which might be due to the serious damage to root vigor under such treatment (see Figure 1 and Table 1).

### 3.4. Exogenous MT Mitigates Oxidative Stress and Maintains Membrane Integrity

**Figure 4:**
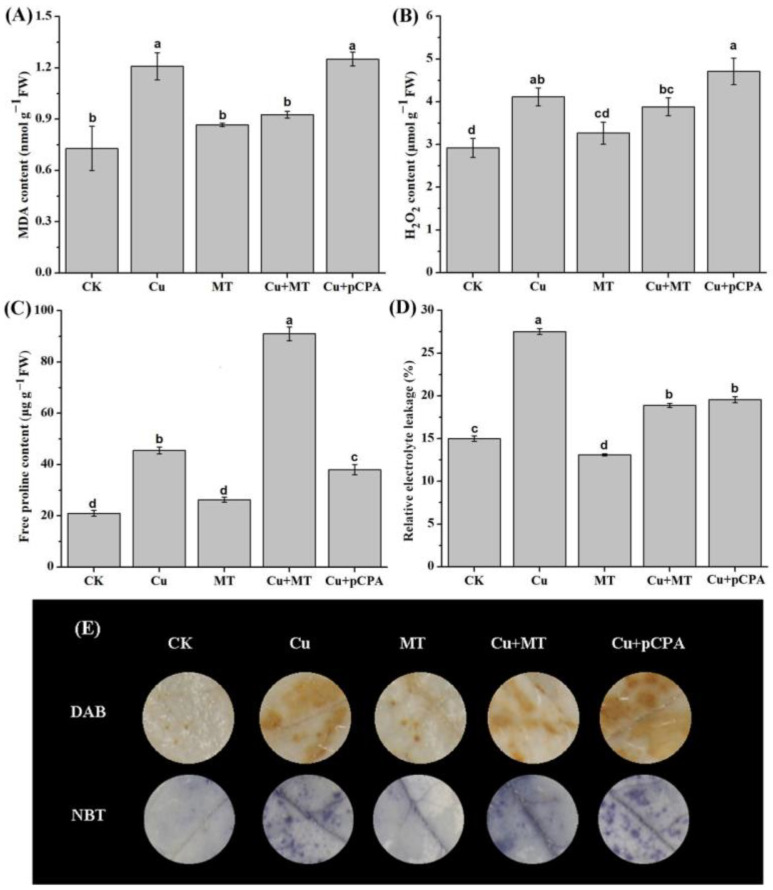
Accumulation of (**A**) MDA; (**B**) H_2_O_2_; (**C**) Free proline; (**D**) Relative electrolyte leakage (REL); (**E**) Histochemical staining by 3,3-diaminobenzidine (DAB) of H_2_O_2_, and nitroblue tetrazolium (NBT) of superoxide radicals (O_2_^•−^). CK, ordinary culture solution; Cu, culture solution containing 0.1 mM Cu; MT, ordinary culture with folia application of 0.1 mM MT; Cu + MT, ordinary culture solution containing 0.1 mM Cu with folia application of 0.1 mM MT; Cu + pCPA, ordinary culture solution containing 0.1 mM Cu with 0.1 mM MT synthesis inhibitor pCPA. The various letters (a, b, c, d, ab, bc, cd) specify considerable discrepancies for *p* values lower than 0.05 based on Duncan’s multiple scope tests.

To explore the influence of exogenous MT in managing plasma membranes’ integrity against the action of CS, malondialdehyde (MDA), proline, and REL were appropriately measured (see Figure 4A,C,D). Compared with the CK group, MDA, proline, and REL grew by 65.89%, 116.6%, and 83.53%, respectively, within the leaves subjected to the Cu treatment. The Cu + pCPA treatment led to growth of the aforementioned three factors by 3.5%, 16.5% and 28.91% in comparison with the Cu treatment. Furthermore, exogenous MT reduced the abovementioned three parameters by approximately 23.42%, 99.9% and 31.38%, respectively, in comparison with the individual Cu treatment.

Furthermore, the influence of MT in lessening Cu-induced oxidative injury was evaluated. The leading plant biomarker of oxidative damage, H_2_O_2_, was measured in the leaves of tomato subjected to CS. (see Figure 4B). The results shown in Figure 4B reveal that Cu treatment led to a substantial increase in the H_2_O_2_ concentration, by 41%, in the leaves of tomato seedlings in comparison with CK. Meanwhile, the MT treatment reduced the average content of H_2_O_2_ (by 5.6%) in comparison with that in plants acted upon by CS (see Figure 4B). In order to visualize how ROS (H_2_O_2_ and O_2_^•−^) accumulated in plants under CS, and how MT alleviated such an effect, we stained the leaves of tomato plants with DAB and NBT (see Figure 4E). Compared with CK, the leaves of the plants acted upon by Cu demonstrated dark brown (H_2_O_2_) and navy (O_2_^•−^) colors. By comparison, MT treatment lessened the deepness of the DAB and NBT staining within tomato leaves in the presence of CS.

### 3.5. Influence of MT on Enzymatic Antioxidant Capacity in Tomato Leaves under CS

**Figure 5:**
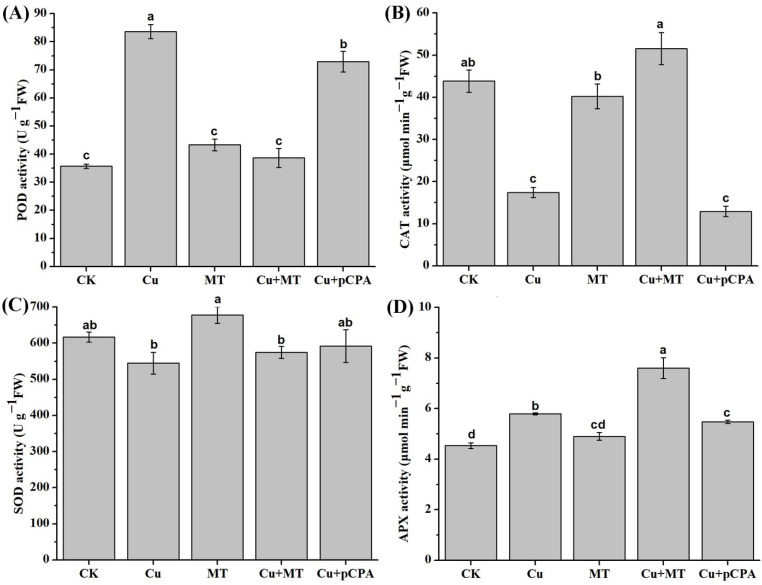
Variations in enzymatic antioxidant activities in tomato leaves by Cu, pCPA, and MT treatments: (**A**) POD; (**B**) CAT; (**C**) SOD; and (**D**) APX. CK, ordinary culture solution; Cu, culture solution containing 0.1 mM Cu; MT, ordinary culture with folia application of 0.1 mM MT; Cu + MT, ordinary culture solution containing 0.1 mM Cu with folia application of 0.1 mM MT; Cu + pCPA, ordinary culture solution containing 0.1 mM Cu with 0.1 mM MT synthesis inhibitor pCPA. The various letters (a, b, c, d, ab, cd) specify considerable discrepancies for *p* values lower than 0.05 based on Duncan’s multiple scope tests.

Plants have evolved a complicated antioxidant system, including antioxidant enzymes and non-enzymatic compounds, to manage oxidative stress. In order to examine the influence of MT on the antioxidant system, the activities of typical antioxidant enzymes, such as SOD, POD, CAT, and APX, were inspected. In the carried-out testing, Cu treatment led to an increase in the activities of POD and APX by 134% and 27.69%, respectively; however, the activities of CAT and SOD decreased by 60.28% and 11.71%, respectively, in comparison with CK (see Figure 5). Exogenous MT treatment in CS-based plants demonstrated additional growth of the enzymatic activities by 195.9%, 5.4%, and 31.26% for CAT, SOD, and APX, respectively (see Figure 5). However, POD in plants subjected to Cu + MT conditions did not rise in comparison with Cu plants. The activities of the POD, CAT and APX decreased by 12.79%, 25.85% and 5.4%, respectively, in the leaves, in the presence of Cu + pCPA in comparison with Cu plants. The obtained results demonstrate that exogenous MT stimulated the mitigation of oxidative stress, which is essentially associated with the upregulation of enzymatic antioxidant processes in tomato plants.

### 3.6. Effect of MT on Nonenzymatic and Total Antioxidant Activity Subjected to CS

**Figure 6:**
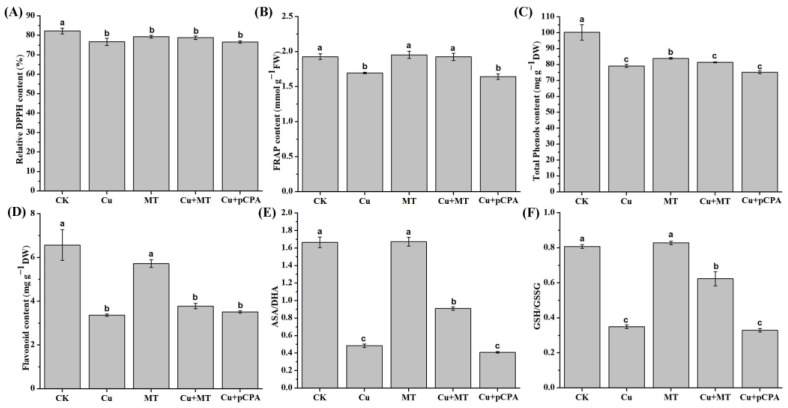
Variations in nonenzymatic and total antioxidant processes in tomato leaves by Cu, MT and pCPA treatments: (**A**) 1,1-diphenyl-2-picrylhydrazyl (DPPH) assay; (**B**) Ferric reducing antioxidant power (FRAP); (**C**) Total phenolics; (**D**) Total flavonoids; (**E**) The ratio of glutathione (GSH) and glutathione disulfide (GSSG); (**F**) The ratio of ascorbic acid (AsA) to dehydroascorbate (DHA). The various letters (a, b, c) specify considerable discrepancies for *p* values lower than 0.05 based on Duncan’s multiple scope tests.

For the purpose of examining the effect of MT in nonenzymatic compounds, the total phenolic and flavonoid contents in tomato leaves were appropriately estimated and the total antioxidant capacity was determined using FRAP and DPPH assays. Only some of the antioxidant compounds and capacities exhibited significant differences in these treatments. For example, the content of DPPH showed no change, while FRAP grew by 13.59% when comparing Cu + MT plants with Cu plants (see Figure 6A,B). As demonstrated in Figure 6C,D, the total phenolics and flavonoids neither increased nor decreased in the Cu + MT- or Cu + pCPA-treated plants, compared with plants under the action of only Cu treatment.

Compared with Cu-treated plants, the GSH/GSSG and AsA/DHA ratios within Cu + MT-treated plants increased by 245.4% and 137%, respectively, whereas the ratios were decreased in Cu + pCPA plants (see Figure 6). Generally, these discoveries provide valuable clues that exogenous MT could have an encouraging impact on the alleviation of oxidative stress, which may be associated with the boosting of nonenzymatic antioxidant activities in tomato plants.

### 3.7. Influence of Exogenous MT on Gene Expression Related to Detoxification and MT Biosynthesis

In order to investigate whether MT changes gene expression related to detoxification and MT biosynthesis in plants, we analyzed the transcriptional levels of ROS detoxification-related genes, such as monodehydroascorbate reductase (MDHAR), dehydroascorbate reductase (DHAR), and glutathione reductase (GR). The expression levels of detoxification-related genes (*SOD*, *CAT*, *APX*, *GR*, and *MDHAR*) were upregulated in Cu + MT-treated plants, compared with Cu plants. The obtained results imply that exogenous MT could decrease Cu toxicity by inducing gene expression of these ROS detoxification-related genes.

**Figure 7:**
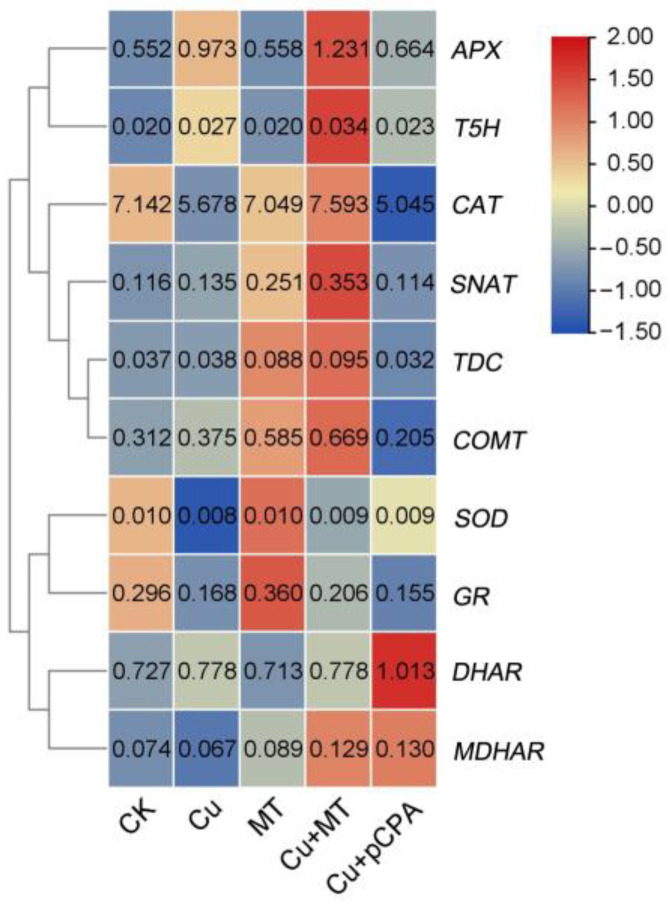
Heat-map demonstrating the relative transcript abundance of differentially expressed antioxidant enzyme-encoding genes and MT biosynthesis genes in leaves of tomato seedlings subjected to CS; hierarchical cluster analysis also employed. The heat-map was generated with the Tbtools software [50]. Expression across each gene (or row) is scaled by normalization. The number in each square of the heat-map indicates the relative expression values for each gene. Monodehydroascorbate reductase, *MDHAR*; dehydroascorbate reductase, *DHAR*; glutathione reductase, GR; catalase, *CAT*; ascorbate peroxidase, *APX*; superoxide dismutase activity, *SOD*; tryptophan decarboxylase, *TDC*; tryptamine 5-hydroxylase, *T5H*; serotonin N-acetyltransferase, *SNAT*; caffeic acid O-methyltransferase, *COMT*.

Tryptophan decarboxylase (*TDC*), tryptamine 5-hydroxylase (*T5H*), serotonin N-acetyltransferase (*SNAT*) and caffeic acid O-methyltransferase (*COMT*) play important roles in MT biosynthesis [49]. The foliar application of MT caused an increase in the expression of these genes (except for *T5H*), compared with CK (see Figure 7). Moreover, the expression of these genes was higher with Cu + MT treatment compared with Cu. Overall, these results suggest that MT biosynthesis is induced by, and plays an important role in alleviating, Cu toxicity in tomato plants.

## 4. Discussion

Cu, an essential cofactor of electron transport chain components in mitochondria and chloroplasts, is important for many processes in plants [3]. However, several important sources, such as fertilizers, sewage sludge, pesticide application, agrochemicals and industrial waste, have resulted in Cu over-accumulation in soil and water. Unfortunately, this over-accumulation can have detrimental effects upon and directly influence plant growth and development, which ultimately affects crop yield and quality [7]. Recently published reports suggested that MT has shown a protective role against HM [29,30]. However, MT has not been examined yet in tomato with respect to Cu stress. The aim of our study was to investigate the function of MT on overcoming Cu toxicity.

pCPA is an irreversible tryptophan hydroxylase inhibitor known to diminish MT biosynthesis, which has been well-studied in animals [51,52,53]. However, it is barely studied in plants. In the present work, we produced MT-deficient tomato plants by applying pCPA and via the silencing of a gene (*COMT1*) that is critical for MT biosynthesis. Through combining MT-rich plants and MT-deficient plants, more reliable results could be obtained when studying the function of MT on Cu-induced stress. The results showed that pCPA application severely affected the plant growth and root vitality. COMT plays an important role in MT biosynthesis in Arabidopsis and rice [54,55]. In tomato seedlings, cadmium stress activates the transcription of *COMT1*, thereby encouraging the accumulation of MT and increasing cadmium tolerance [56]. In tomato, it has been revealed that silencing of *COMT1* intensifies heat stress by hindering both the light reactions and the carbon fixation reactions of photosynthesis, and MT is effectively suppressed [49].

Our study showed that CS causes substantial harmful influences at the morphological level. Regarding these morphological aspects, the most obvious phenotypic change under CS was plant dwarfing. The destructive effects on tomato growth are in good agreement with previous studies of HM toxicity in other crops, including maize, wheat, and mustard [7,57,58]. It is worth mentioning that root vitality was remarkably affected by CS, which decreased by 80% under CS in comparison with the CK plants. Roots not only provide structural support to the aerial parts of plants, but also supply nutrients and water. Thus, a plant’s survival depends on its appropriate growth, development, and root function. However, CS severely impairs root growth, and this damage can be alleviated by MT application. Leaf chlorosis was not visible until after four weeks of treatment in CS (see Figure 1B and Supplementary Figure S2). Excess Cu was found to reduce photosynthetic pigment content, photosynthesis and transpiration rates and Fv/Fm in plants [59,60]. Therefore, the tricarboxylic acid (TCA) cycle and PSII (photosystem II) reaction were damaged by CS, which led to plant dwarfing and reduced ability to mitigate heavy metal damage. Conversely, MT supplementation notably enhanced plant growth. At the same time, the ultrastructure of mesophyll cells and chloroplasts showed that Cu stress disturbs the order and shape of cells. MT treatment lessened such an injury to the ultrastructure, as explained and discussed in previous investigations [61,62]. We found that starch grains appeared in the mesophyll cells after MT application. A previous study reported that MT improved waxy maize seed germination under chilling stress through improving the antioxidant system and starch metabolism [63]. Therefore, we suspect that MT could protect the mesophyll cells by regulating starch metabolism; the transcriptome and metabolome are in progress, and more attention should be paid to starch metabolism-related genes and substances.

Under CS, plants induce the production of ROS, which cause oxidative stress [64]. In general, the well-known function of MT is that it scavenges excessive ROS and maintains redox homeostasis in the presence of environmental stresses [25]. ROS results in oxidative stress and disorder of the redox balance in plants. The membranes of cells, nucleic acids, proteins, and numerous physiological procedures can be damaged by oxidative stress [8,65,66,67]. In our study, through testing H_2_O_2_ content and the distribution of H_2_O_2_ and O_2_^•−^, we found that Cu treatment accelerated the production of H_2_O_2_ and O_2_^•−^, and such production was further increased by the application of pCPA (Figure 4B,E). Exogenous MT application could boost endogenous MT accumulation and reduce the production of H_2_O_2_ (Figure 3A and Figure 4B). Conversely, pCPA was proved to inhibit MT accumulation and increase the generation of ROS (Figure 4B,E). Correspondingly, over-accumulation of ROS was closely associated with higher contents of MDA and REL, which reduced the integrity of the cellular membrane, indicating that Cu stress caused severe oxidative damage in tomato seedlings (Figure 4A,D). MT application reduced the excess generation of H_2_O_2_ and O_2_^•−^, along with lowering the levels of MDA and REL, which indicated that MT might alleviate Cu-induced oxidative stress and thus allow tomato plants a better growth environment under Cu toxicity. Our results are consistent with some earlier reports on other plants that were tested under stress from other heavy metals [15,29]. Proline is an important osmotic adjustment substance in plants and maintains the reliability of the membrane structure, which can be used as a physiological and biochemical indicator for plants to respond to stress. It is confirmed that plants can accumulate free proline when subjected to HM stress [15]. In the present study, the free proline content of leaves increased under the action of CS, and exogenous MT further increased the proline accumulation in tomato in the presence of Cu-induced stress. Zhao et al. (2017) reported that exogenous MT causes an increase in the accumulation of proline in gardenia plants under the action of darkness-induced stress [62]. Overall, our results indicate that the exogenous application and endogenous accumulation of MT play an important role in scavenging ROS production and protecting membrane integrity in tomato plants under CS.

Plants have established various mechanisms to defend themselves to respond to HMs. For many plants, the first line of defense is to avoid or lessen the acceptance of HMs into root cells, which could be achieved by limiting them to the apoplast, attaching them to the cell wall, or inhibiting long distance transport. If this does not work, plants employ a range of storage and detoxification methods, such as metal transportation, chelation, trafficking, and sequestration into the vacuole [68]. Jahan et al. [29] found that MT alleviates nickel phytotoxicity by reducing the nickel accumulation in plants. A previous study reported that MT alleviated Cu toxicity by improving Cu sequestration, rather than influencing Cu uptake, in cucumber [30]. However, in this study, there was no significant difference in Cu accumulation with or without MT application (Figure 3B). At the same time, a high content of MT did not reduce Cu accumulation in the roots, but increased the content of Cu in the shoots (Figure 3B). These results demonstrate that MT may act differently under conditions of different metals and plants, which requires more in-depth study in the future. The mechanism by which MT alleviates Cu stress is not by reducing the absorption and transport of Cu, but through other systems, such as the antioxidative system. When the methods displayed above do not work effectively, plants induce oxidative stress defense mechanisms, including the synthesis of stress-related proteins and signaling molecules (i.e., heat shock proteins, hormones, and ROS). If the antioxidant capacity of a plant under HM stress is not strong enough, then the application of exogenous compounds with high antioxidant properties could be helpful to increase the plant’s tolerance to a particular stress [69]. MT, which has antioxidant properties, is reported to have a remarkable influence on plants’ response to numerous abiotic stresses, such as heat/cold stress, drought, salinity and HM toxicity [15,20,21,27].

Plants use antioxidant defense systems to alleviate the oxidative damage from excess Cu accumulation in cells. These include enzymatic and nonenzymatic scavenging systems. The main mechanism for scavenging ROS is the enzymatic system, which can significantly affect the ability of plants to adapt to unfavorable environments. The enzymes in the enzymatic scavenging system mainly include SOD, POD, CAT, APX, etc. SOD is considered the first key line of defense to remove active oxygen in plants. SOD can scavenge O_2_^•−^ and generate H_2_O_2_. Subsequently, H_2_O_2_ can be degraded and removed by APX and CAT. During Cu-induced stress, MT application significantly increased the activity of APX and CAT, but decreased the activity of POD. The SOD activity did not change a lot in all of the treatments; this could be explained by the fact that the sampling time was two weeks after CS, and as the first line of defense, SOD already played its role in O_2_^•−^ scavenging, which in turn resulted in H_2_O_2_ production. In our study, the gene expression of *CAT* was much higher than that of *APX*. Compared with APX, CAT had higher enzymatic activity, but weaker affinity. CAT and APX are key enzymes in the removal of H_2_O_2_ in plants, and APX had a high affinity. CAT usually works when the concentration of H_2_O_2_ is high, while the scavenging effect at low concentrations of H_2_O_2_ is not obvious. Generally, MT mitigates Cu toxicity to tomato seedlings by enhancing the enzymatic scavenging system, especially via APX and CAT to remove H_2_O_2_, but not SOD or POD to remove O_2_^•−^.

The effect of MT on nonenzymatic compounds was also determined. Derivatives of phenolic compounds are secondary metabolites produced by plants. One of the largest groups of phenolic compounds is the flavonoid group. A previous study revealed that MT might play a role in the alleviation of oxidative stress by stimulating phenol and flavonoid compounds to scavenge ROS [70]. Interestingly, in our study, the total phenolic and flavonoid contents increased slightly, but there was no significant difference in Cu + MT treatment compared with Cu treatment. It could be deduced that the phenols and flavonoids varied simultaneously and were interdependent to some extent, and thus did not correlate well to MT application. MT does not alleviate copper stress by inducing total phenolic and flavonoid contents. Antioxidant activity was tested using the DPPH and FRAP methods. The principle of the DPPH method is that compounds containing antioxidant activity will react with DPPH through electron donors from antioxidant compounds to DPPH [71]. It can measure antioxidant activity quickly, simply, and inexpensively. This study also measured antioxidant activity using the FRAP method, which uses a different principle from the DPPH method, this principle being the reduction of the ferric tripyridyltriazine (Fe(III)-TPTZ) complex to ferric tripyridyltriazine (Fe(II)-TPTZ) by antioxidants at low pH [72]. In our study, the results from the two methods were different; in the FRAP test antioxidant activity was significantly induced in Cu + MT plants compared with Cu plants, while in the DPPH test there was no difference. This could be due to the long period between testing with the DPPH and FRAP methods, so that there were differences in the sample conditions. Moreover, different types of substances may obtain different results using the FRAP method and the DPPH method, because the FRAP method focuses on the detection of antioxidant reduction ability while the DPPH method focuses on free radicals.

The AsA/GSH cycle was entirely taken into account as a scavenging procedure, mostly and expertly eliminating the ROS. The ratio of GSH to GSSG, a powerful index of oxidative stress, is important for redox homeostasis in plants [73]. AsA and its oxidized form DHA are crucial for redox state-based signaling mechanisms via the detoxification of ROS and their products, as well as the transmission of redox signals [74]. DHA is the oxidized form of AsA, and both are involved in redox state-based signaling mechanisms via erasing of ROS and their products, as well as carrying redox signals [75]. Glutathione, which is a member of the AsA–GSH cycle, participates in the defense against ROS. Glutathione exists in two forms, oxidized glutathione (GSSG) and reduced glutathione (GSH), and the reduction potential of GSH depends on the intracellular GSH/GSSG ratio. In this study, we found that CS lessened the GSH/GSSH and AsA/DHA ratios, which is in agreement with studies in many crops subjected to HM stress, e.g., arsenic stress in maize [76] and copper stress in rice [77]. Our obtained results also revealed that when MT was added, the GSH/GSSH and AsA/DHA ratios increased significantly in the roots compared with Cu^2+^ treatment alone. In AsA metabolism, APX uses AsA as an electron donor in the catalytic reaction to eliminate H_2_O_2_; MDHAR catalyzes monodehydroascorbic acid (MDHA) to AsA; DHAR catalyzes GSH and converts DHA to AsA; and GR can catalyze the reduction of GSSG to GSH, thereby keeping the intracellular GSH pool in a reduced state. In our study, transcript expression of *APX*, *MDHAR* and *GR* was induced after MT application under CS treatment; however, the level of DHAR remained unchanged. Therefore, MT is mainly involved in the regulation of oxidation injury under CS through improving the expression of *APX* to eliminate H_2_O_2_, *GR* to maintain the intracellular GSH pool, and *MDHAR* instead of *DHAR* to maintain the level of AsA. Taken together, our results reveal that MT alleviates Cu toxicity by ROS scavenging, enhancing antioxidant capacity when plants are subjected to excessive Cu.

## 5. Conclusions

By employing MT-rich and MT-deficient tomato plants, we exhibited the mechanism by which MT alleviates CS in tomato. MT is effective in the reduction of oxidative stress caused by Cu^2+^, and the evidence that we presented includes: (1) the mechanism by which MT alleviates Cu stress is not by reducing the absorption and transport of Cu, but mainly through improving the antioxidative system; (2) MT leads to the lessening of ROS accumulation and protects the cell membranes in Cu-stressed tomato leaves; (3) MT enhances the activities of antioxidant enzymes, especially APX and CAT; (4) MT induces the gene expression of detoxification-related genes and MT biosynthesis-related genes. Generally, MT is a key factor for the manipulation of Cu^2+^ tolerance in tomatoes, and it can be employed as a new tactic to defend against the damage caused by CS. In the next step, an in-depth molecular approach is needed to study the mechanisms of MT-meditated enhanced tolerance to Cu in plants.

## Figures and Tables

**Table 1:** Variations in growth, root vigor and chlorophyll resulting from various treatments.

Treatment Parameter	CK	Cu	MT	Cu + MT	Cu + pCPA
Plant height (cm)	17.71 ± 0.49b	11.62 ± 0.30c	22.08 ± 0.60a	15.45 ± 0.14d	7.84 ± 0.25e
Shoot Fresh Weight (g/plant)	15.92 ± 0.79b	9.33 ± 0.37d	26.25 ± 1.38a	13.04 ± 0.80c	5.58 ± 0.33e
Root Fresh Weight (g/plant)	3.24 ± 0.13b	2.44 ± 0.10c	4.71 ± 0.41a	4.61 ± 0.27a	1.57 ± 0.10d
Shoot Dry Weight (g/plant)	1.45 ± 0.06b	1.07 ± 0.02c	1.92 ± 0.08a	1.41 ± 0.03b	0.61 ± 0.01d
Root Dry Weight (g/plant)	0.37 ± 0.01c	0.32 ± 0.01d	0.52 ± 0.01a	0.44 ± 0.01b	0.2 ± 0.01e
TTC [ug/(g·h)]	4547.92 ± 150.91b	170.56 ± 20.41c	7225.19 ± 401.94a	733.95 ± 27.75c	151.23 ± 8.96c
Total Chlorophyll (C_a_ + C_b_) (mg/g·FW)	1.77 ± 0.12b	0.80 ± 0.03d	2.07 ± 0.08a	1.43 ± 0.05c	0.74 ± 0.10d

Various letters specify substantial discrepancies at *p* < 0.05 based on Duncan’s multiple range tests. Root vigor was tested using triphenyltetrazolium chloride (TTC). CK, ordinary culture solution; Cu, culture solution containing 0.1 mM Cu; MT, ordinary culture with folia application of 0.1 mM MT; Cu + MT, ordinary culture solution containing 0.1 mM Cu with folia application of 0.1 mM MT; Cu + pCPA, ordinary culture solution containing 0.1 mM Cu with 0.1 mM MT synthesis inhibitor pCPA.

## Data Availability

Data are contained within the article and supplementary material.

## Supplementary Materials

The following supporting information can be downloaded at: https://www.mdpi.com/article/10.3390/antiox11040758/s1, Figure S1: Phenotypes after 14 d of Cu^2+^ stress and melatonin and pCPA treatment. Cu, culture solution containing 0.1 mM Cu; MT, ordinary culture with folia application of 0.1 mM MT; Cu + MT, containing 0.1 mM Cu with folia application of 0.1 mM MT; Cu + pCPA, containing 0.1 mM Cu with 0.1 mM MT synthesis inhibitor pCPA. Figure S2: Fv/Fm of leaves under different treatments. Cu, culture solution containing 0.1 mM Cu; MT, ordinary culture with folia application of 0.1 mM MT; Cu + MT, containing 0.1 mM Cu with folia application of 0.1 mM MT; Cu + pCPA, containing 0.1 mM Cu with 0.1 mM MT synthesis inhibitor pCPA. Table S1: The gene and primer sequences used for RT-PCR. Monodehydro ascorbate reductase, MDHAR; dehydro ascorbate reductase, DHAR; catalase, CAT; ascorbate peroxidase, APX; superoxide dismutase activity, SOD; tryptophan decarboxylase, TDC; tryptamine 5-hydroxylase, T5H; serotonin N-acetyltransferase, SNAT; caffeic acid O-methyltransferase, COMT.
