# Supplementary material for: Melatonin Alleviates Copper Toxicity via Improving ROS Metabolism and Antioxidant Defense Response in Tomato Seedlings
Source: Antioxidants (Basel). 2022 Apr 11;11(4):758. doi: 10.3390/antiox11040758 (PMC9025625; doi:10.3390/antiox11040758)
Supplement: Supplementary file 1 [file antioxidants-11-00758-s001.zip › antioxidants-1609812-supplementary.pdf]

Supplementary Materials

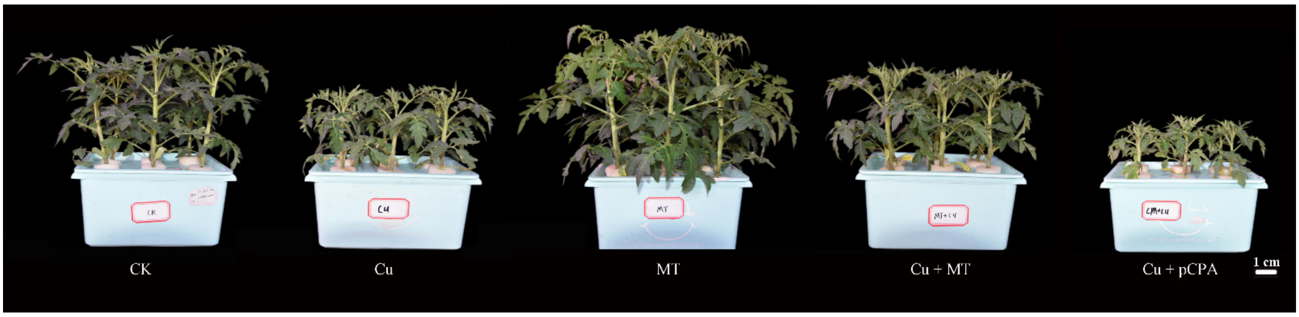

Figure S1. Phenotypes after 14 d of Cu^{2+} stress and melatonin and pCPA treatment. Cu, culture solution that contained 0.1 mM Cu; MT, culture solution with folia application of 0.1 mM MT; Cu+MT, culture solution containing 0.1 mM Cu with folia application of 0.1 mM MT; Cu+pCPA, culture solution containing 0.1 mM Cu with 0.1 mM MT synthesis inhibitor pCPA.

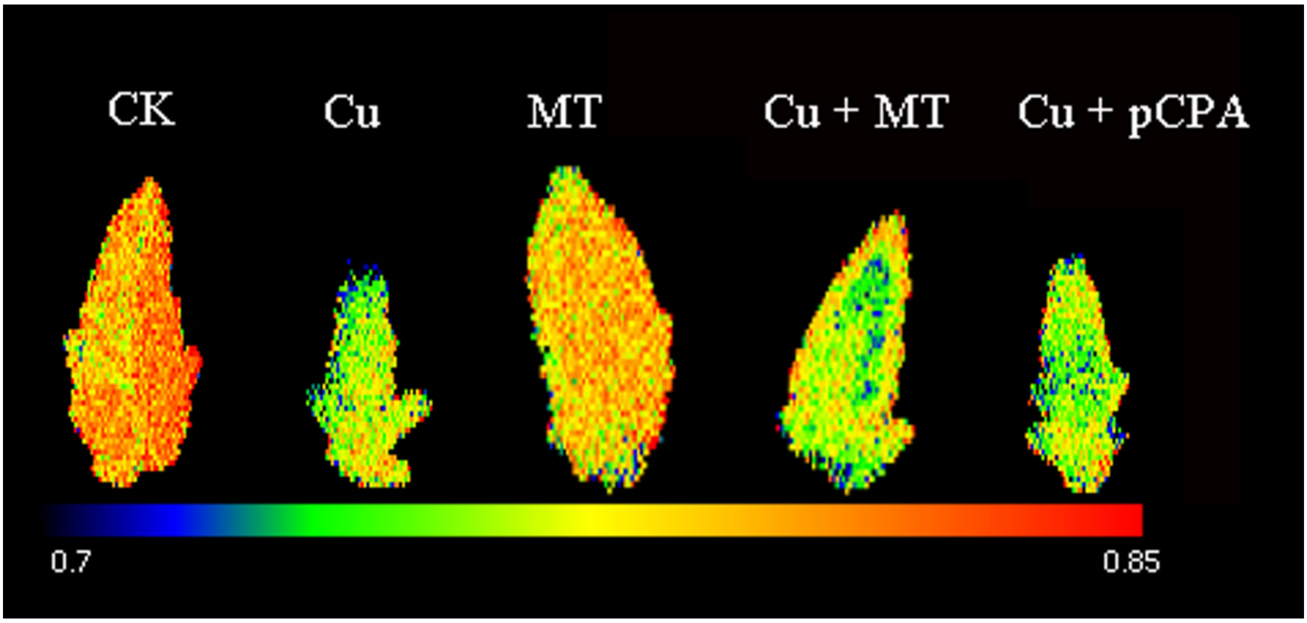

Figure S2. Fv/Fm of leaf under different treatment. Cu, culture solution that contained 0.1 mM Cu; MT, ordinary culture solution with folia application of 0.1 mM MT; Cu+MT, culture solution containing 0.1 mM Cu with folia application of 0.1 mM MT; Cu+pCPA, culture solution containing 0.1 mM Cu with 0.1 mM MT synthesis inhibitor pCPA.. .

Table S1. The primer sequences used in qRT-PCR and VIGS vector construction. Superoxide dismutase activity, SOD; catalase, CAT; ascorbate peroxidase, APX; glutathione reductase, GR; monodehydro ascorbate reductase, MDHAR; dehydro ascorbate reductase, DHAR; tryptophan decarboxylase, TDC; tryptamine 5-hydroxylase, T5H; serotonin N-acetyltransferase, SNAT; caffeic acid O-methyltransferase, COMT.

Gene full name	Gene Acronym	Gene Accession No.	Forward Primer	Reverse Primer	Comment
Superoxide dismutase	SOD	XM_004232522	ACTACTCCCAGTTGCATCCC	CACCAGGAGCAGCCATGATA	qRT-PCR
Catalase	CAT	NM_001247898	GCAGCTCCCAGTTAATGCTC	AGCAGGACGACAAGGATCAA	qRT-PCR
Ascorbate peroxidase	APX	NM_001247859	GGCACTCTGCTGGTACCTAT	GGAGAGAGTGGGAAACTGCT	qRT-PCR
Glutathione reductase	GR	NM_001247314	GGAGCCATAGAGGTTGACGA	CTCCTCCCTCCATCAAAGCA	qRT-PCR
Monodehydroascorbate reductase	MDHAR	NM_001247084	CGGACAGTTCCGAACAAACA	CCCGTGCAATTCGGTTGTAT	qRT-PCR
Dehydroascorbate reductase	DHAR	NM_001247893	GAGGTGAACCCTGAAGGGAA	CCCACAGAGGCAAATTCAGG	qRT-PCR
Tryptophan decarboxylase	TDC	XM_010328040	CAGTGCCGGGATTAACATGG	GCAAGCCAATCCAGAACGAT	qRT-PCR
Tryptamine 5-hydroxylase	T5H	NM_001247918	CTCCGTCTCCACCATCTCTC	CGGAATTTGGCCGAGTTGAA	qRT-PCR
Serotonin N-acetyltransferase	SNAT	XM_004248987	TAAGGTTGGTTGGCCTCGAA	GGCCATGCCTATCAGCTTCT	qRT-PCR
Caffeic acid *O*-methyltransferase	COMT	XM_004234980	CCGATCACCACCACCTTATCC	CCACCAACATCAACAATGGA	qRT-PCR
Actin	Actin	NM_001321306	TGTCCCTATTTACGAGGGTTATGC	AGTTAAATCACGACCAGCAAGAT	qRT-PCR
Caffeic acid *O*-methyltransferase	COMT1-TRV2	XM_004234980	AAGGTTACCGAATTCTCTAGACTCAAACAGAGGATGAAGC	GAGACGCGTGAGCTCGGTACATAAAGCCGCTCTACACGTC	Vector construction
